# High-throughput transcriptomics reveals common and strain-specific responses of human macrophages to infection with *Mycobacterium abscessus* Smooth and Rough variants

**DOI:** 10.1186/s12864-015-2246-1

**Published:** 2015-12-09

**Authors:** Anna Aulicino, Adam M. Dinan, Aleksandra A. Miranda-CasoLuengo, John A. Browne, Kévin Rue-Albrecht, David E. MacHugh, Brendan J. Loftus

**Affiliations:** School of Medicine & Medical Science, Conway Institute of Biomolecular and Biomedical Research, University College Dublin, Belfield, Dublin 4 Ireland; Animal Genomics Laboratory, UCD School of Agriculture and Food Science, College of Agriculture, Food Science and Veterinary Medicine, University College Dublin, Belfield, Dublin 4 Ireland; UCD Conway Institute of Biomolecular and Biomedical Research, University College Dublin, Dublin 4, Dublin, Ireland

**Keywords:** *Mycobacterium abscessus*, Smooth, Rough, Macrophage, RNA-seq, Transcriptomics, Cytokine, Chemokine, LAMP-3

## Abstract

**Background:**

*Mycobacterium abscessus* (MAB) is an emerging pathogen causing pulmonary infections in those with inflammatory lung disorders, such as Cystic Fibrosis (CF), and is associated with the highest fatality rate among rapidly growing mycobacteria (RGM). Phenotypically, MAB manifests as either a Smooth (MAB-S) or a Rough (MAB-R) morphotype, which differ in their levels of cell wall glycopeptidolipids (GPLs) and in their pathogenicity in vivo. As one of the primary immune cells encountered by MAB, we sought to examine the early transcriptional events within macrophages, following infection with both MAB-S or MAB-R.

**Results:**

We sampled the transcriptomes (mRNA and miRNA) of THP-1-derived macrophages infected with both MAB-R and MAB-S at 1, 4 and 24 h post-infection (hpi) using RNA-seq. A core set of 606 genes showed consistent expression profiles in response to both morphotypes, corresponding to the early transcriptional response to MAB. The core response is type I Interferon (IFN)-driven, involving the NF-κB and MAPK signaling pathways with concomitant pro-inflammatory cytokine production, and network analysis identified *STAT1*, *EGR1*, and *SRC* as key hub and bottleneck genes. MAB-S elicited a more robust transcriptional response at both the mRNA and miRNA levels, which was reflected in higher cytokine levels in culture supernatants. The transcriptional profiles of macrophages infected with both morphotypes were highly correlated, however, and a direct comparison identified few genes to distinguish them. Most of the induced miRNAs have previously been associated with mycobacterial infection and overall miRNA expression patterns were similarly highly correlated between the morphotypes.

**Conclusions:**

The report here details the first whole transcriptome analysis of the early macrophage response to MAB infection. The overall picture at the early stages of macrophage infection is similar to that of other mycobacteria, reflected in a core type I IFN and pro-inflammatory cytokine response. Large-scale transcriptional differences in the host response to the different MAB morphotypes are not evident in the early stages of infection, however the subset of genes with distinct expression profiles suggest potentially interesting differences in internal trafficking of MAB within macrophages.

**Electronic supplementary material:**

The online version of this article (doi:10.1186/s12864-015-2246-1) contains supplementary material, which is available to authorized users.

## Background

*Mycobacterium abscessus* (MAB) is an emerging human pathogen, responsible for a majority of pulmonary infections caused by rapidly growing mycobacteria (RGM) in the United States, with a much higher fatality rate than any other RGM [1]. MAB lung infections usually, but not exclusively, develop in subjects with underlying lung disorders including cystic fibrosis (CF) [2]. In patients with CF, MAB causes a serious, life-threatening lung disease with disseminated, often fatal infections following lung transplantation [2]. MAB is also amongst the most antibiotic-resistant RGM species, with a diverse chromosomally-encoded resistome, and infection often requires prolonged treatment [3].

Phenotypically, MAB manifests as either a Smooth (MAB-S) or a Rough (MAB-R) colony morphotype. The transition from MAB-S to MAB-R is associated with the loss of glycopeptidolipids (GPLs) in the outermost layer of the cell envelope. GPLs confer several surface phenotypes, such as aggregation, sliding motility and biofilm formation [4] and, depending on structural modifications, may be important immunomodulators [5–7] or inert molecules [6, 8, 9]. MAB-S is reported to retain the ability to form biofilms and to colonize surfaces but is unable to cause persistent infections, while MAB-R does not form biofilms but invades and multiplies within macrophages, causing persistent infections [9, 10]. The primary objective of this work is to facilitate a greater understanding of the early transcriptional events associated with the host macrophage response to infection with MAB. In addition, we hoped to identify indicative differences in the early host transcriptional responses to the MAB-S or MAB-R variants, to gain insights into their potential contribution towards the progress of infection.

## Results and discussion

### Library generation and clustering

THP-1-derived macrophages were infected in parallel with Smooth and Rough variants of *M. abscessus* ATCC 19977-IP [11, 12]. Samples were collected at 1, 4 and 24 h post-infection (hpi), with the objective of capturing the initial interaction of host and pathogen, the early response of the host and a slightly later response following the establishment of infection. The number of bacteria (CFU) and macrophage viability were assessed at each time point post infection. No significant differences were observed in MAB CFU, and the percentages of dead macrophages were comparable for both strains (Additional file 1: Figure S1 and Additional file 2: Figure S2).

**Fig. 1:**
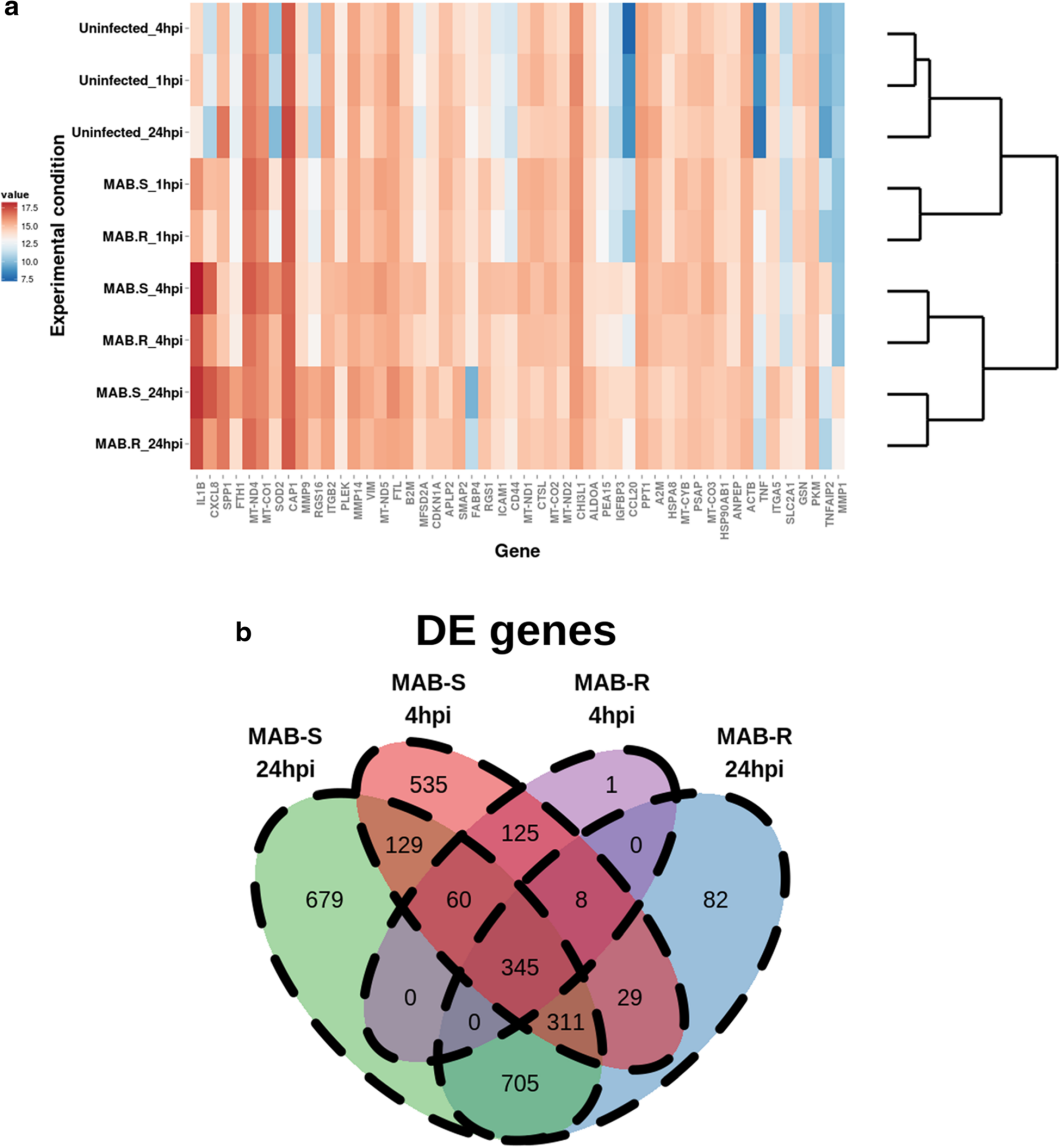
Library clustering and overlap of DE genes. **a** Unsupervised hierarchical clustering of mRNA libraries based on the normalised expression levels of the 50 most variable genes. Libraries from MAB-R- and MAB-S-infected cells group separately from uninfected cells, with the 4 and 24 hpi infection libraries forming a discrete cluster from all 1 hpi libraries. **b** Venn diagrams illustrating the overlap of DE genes identified for mRNA and miRNA libraries at 4 and 24 hpi. The numbers of DE genes were found to increase progressively during infection in both cases. MAB-S elicited a more robust response, with MAB-R inducing comparatively fewer unique DE genes

RNA sequencing (RNA-seq) of poly-A selected messenger RNA (mRNA) and small RNA-seq (sRNA-seq) of size-selected micro-RNA (miRNA) transcripts from infected cells and uninfected controls were performed at 1, 4 and 24 hpi. On average, 18.7 and 0.7 million reads from the RNA-seq and sRNA-seq libraries, respectively, were uniquely mapped to the human genome (Additional file 3: Table S1). In total, 12,656 mRNAs and 395 mature miRNAs could be detected in at least one experimental condition (see Methods). Unsupervised hierarchical clustering showed that samples from infected macrophages grouped distinctly from uninfected controls at each time point (Fig. 1a). Additionally, infected samples from the 4 and 24 hpi time points clustered separately from all 1 hpi samples, indicating clear temporal alterations in gene expression driven by the infection process. Multidimensional scaling (MDS) analysis (Additional file 4: Figure S3) also clearly illustrates the separate clustering of infected and uninfected samples, and their segregation according to time point.

### Differential gene expression

To investigate the differential expression of mRNA transcripts upon infection with either MAB-S or MAB-R, a generalized linear model (GLM) was implemented using edgeR [13], allowing the comparison of controls and infected samples at each time point. Genes with at least a 2-fold change in abundance and with a *P*-value of below 0.05, with multiple testing correction according to the Benjamini-Hochberg approach, were regarded as differentially expressed (DE). At 1, 4 and 24 hpi, we identified 12, 539 and 1480 DE genes, respectively, between MAB-R-infected cells and uninfected controls (Fig. 1b and Additional file 5: Table S2). When comparing MAB-S-infected cells to uninfected controls, 73, 1542, and 2229 such genes were identified, respectively (Fig. 1b and Additional file 5: Table S2). Overall, these figures indicate that the number of DE genes increased progressively during infection, likely reflecting an early re-programming of the host cellular machinery, followed by a more robust transcriptional response upon the adaptation of the bacilli to their host.

### Temporal expression models identify a core response to *MAB* infection

**Fig. 2:**
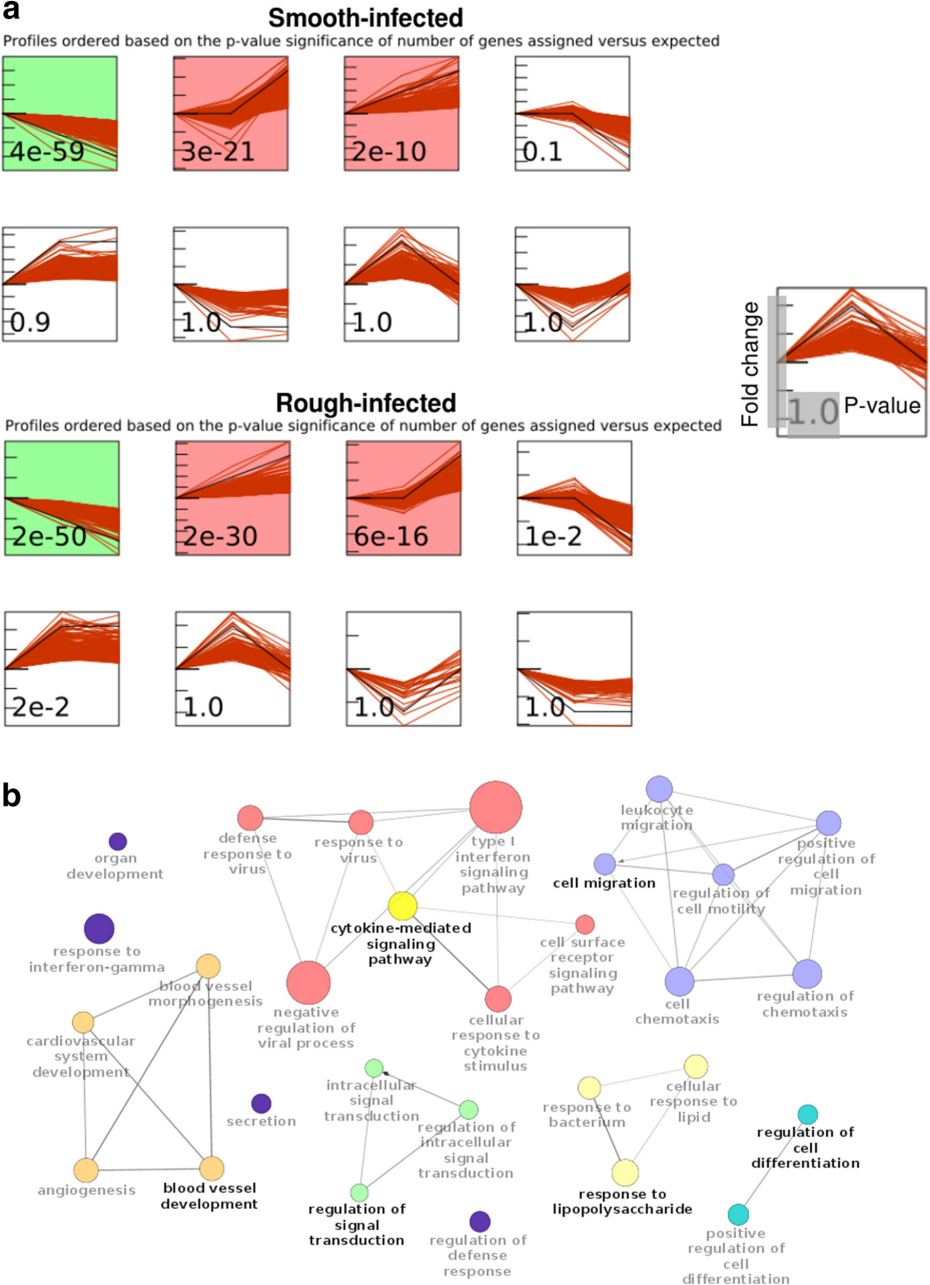
Clustering of core response genes and enriched GO terms. **a** Temporal model profiles of DE genes identified using STEM and ordered by *P* values indicating the significance of enrichment. Profiles shown in color contain a significant over-representation of genes, and the overlap of these profiles comprises the core response. **b** Connectivity among enriched GO terms. Edge size indicates the level of overlap of genes shared between the enriched GO terms, and node size indicates the level of enrichment of the GO term

Given the significant overlap of DE genes between MAB-S and MAB-R infected cells (Fig. 1b), we sought to identify genes with highly similar response profiles. The Short Time Series Expression Miner (STEM) [14] was used to define temporal profiles for all DE genes (Fig. 2a). Profiles with a significant level of overlap between MAB-S- and MAB-R-infected cells (see Methods), comprising a total of 606 genes, were considered to comprise the core response.

GO term enrichment of core response genes indicated a prominent role for the type-I Interferon and cytokine-mediated signaling pathways in the host response to both morphotypes (Table 1). Further analysis using ClueGO [15] demonstrated that many of the enriched GO terms share core response genes in common, and are thus highly interconnected (Fig. 2b).

**Table 1:** GO terms enriched among core response genes. The five most significantly over-represented biological processes identified through GO enrichment analysis of core response genes

Biological process	GO term	Core Response genes in GO term	Total genes in GO term	*P*-value (adj.)
Type-I interferon signaling pathway	GO:0060337	20	63	4.25E-012
Cytokine-mediated signaling pathway	GO:0019221	36	249	2.10E-011
Innate immune response	GO:0045087	96	1359	2.13E-011
Response to virus	GO:0009615	27	140	2.51E-011
Defense response to virus	GO:0051607	25	146	2.50E-009

*M. tuberculosis* (MTB) infection of macrophages induces an IFN response, and the transcriptional signature of THP-1 cells exposed to different MTB strains has been described [16]. In both cases, a large majority of core response genes were found to be upregulated, rather than repressed, upon infection. Seventy three of the core response genes to MAB were also identified as part of the core response to MTB (Additional file 6: Table S3), representing a highly significant overlap (*P* < 5.482e-24, hypergeometric test) and suggesting a conserved role for type-I IFN responses across diverse mycobacterial species. Eighty-two percent of genes overlapping the two datasets were upregulated in both cases, including all IFN-inducible and cytokine-related genes. Just a single gene (*FBP1*) was identified as repressed in both datasets.

The co-expression of genes within the MAB core response suggests that some of these genes may also be co-regulated. To identify possible upstream regulators of the response, we searched for transcription factor binding sites (TFBS) with enrichment proximal to core response genes using the cisRED database [17]. Four TFBSs were identified as having significant enrichment, each of which has a known role in the production of pro-inflammatory cytokines. These included the Interferon Regulatory Factor 1 (IRF-1), a key mediator of the type-I IFN response pathway [18], as well as activator protein-1 (AP-1) [19], the CCAAT/enhancer-binding protein beta (CEBPβ) [20], and nuclear factor erythroid-2 (NFE-2) [21].

### Network analysis of the core response reveals scale-free, small world properties

To examine more closely the correlations between the genes of the core response to MAB infection, we incorporated a network based approach using experimentally-validated interactions from the InnateDB database [22]. The degree (i.e. number of connections) was calculated for each node in the network, and a power law was fitted to the degree distribution of the network. This yielded an R^2^ value of 0.889 and a correlation of 0.882, suggesting that the network has a scale-free architecture, in which a small subset of nodes show a much higher degree of connectivity than the average [23]. This is a common property of cellular networks, in which certain nodes (or genes) show high connectivity and are referred to as hubs [24].

Scale-free biological networks tend to have a high clustering coefficient and a short mean path length between nodes, and are therefore be referred-to as “small-world” networks [25]. Comparing the core response network to 1000 randomly generated networks of an equivalent size (see Methods) revealed that the clustering co-efficient (*C*) of the former (0.102) was ~7.3 times the mean *C* value (0.014) of random networks, while the average shortest path lengths (*L*) were similar (3.913 and 4.738, respectively). These values imply that the core response network has a small-world coefficient (σ) [26] of 8.78 (see Methods), which is similar to that observed for other small-world biological networks [26].

### Key hub and bottleneck genes of the core response network

**Fig. 3:**
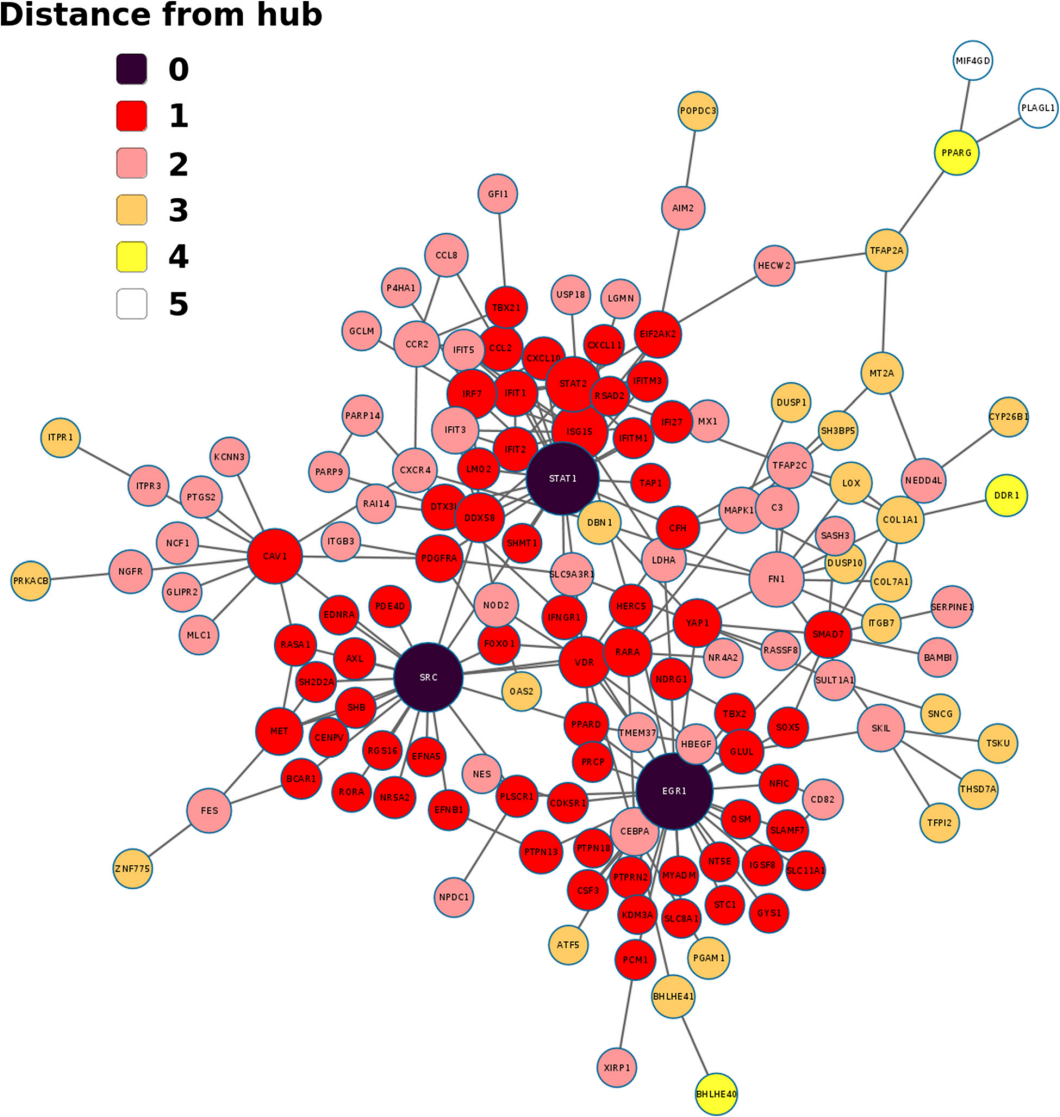
Connectivity of the core response network. Almost all of the genes within the core response (97 %) are linked via a maximum of 3 edges to a hub gene. Edges between genes indicate a validated interaction, node size reflects the degree of connectivity (DOC), and node color indicates the minimum number of edges separating the node from the nearest hub gene

The largest cluster of genes within the core response network consisted of 145 nodes connected via 212 edges (Fig. 3). The genes with highest degrees of connectivity (DOC) and betweenness centrality (BC) were *STAT1*, *SRC*, and *EGR1*, which were thus considered the primary hub and bottleneck nodes. In total, 97 % of genes within the network were linked to one of the 3 hub genes by a maximum of 3 edges (Fig. 3), illustrating the prominence of these genes in mediating the core response. Comparing this result to 1000 randomly re-wired networks demonstrates that this degree of connectivity is significantly higher than would be expected due to chance (*P* < 0.05) (Additional file 7: Figure S4). The BC values of *STAT1*, *SRC*, and *EGR1* were 0.365, 0.351, and 0.315, respectively, which are considerably higher than the network mean (0.02). Similarly, their respective DOC values (27, 24, and 30) were much greater than the mean DOC (3.67) (Additional file 8: Figure S5).

IFN generally potentiates macrophage activation via a *STAT1*-dependent pathway and functional *STAT1* is essential to the host defense against mycobacterial infection [27]. *SRC* is known to be involved in regulating numerous downstream inflammatory signaling pathways and a similar network approach, using MTBC infection, found a central *SRC* hub of the host response network responsible for phagolysosome acidification and the induction of autophagy [28]. Another study using high multiplicity mycobacterial Infection identified *EGR1* downstream of an *SRC*/integrin signaling axis, resulting in the triggering of a NF-kB-mediated NLRP3 inflammasome response [29]. Additionally, a recent study identified *EGR1* as part of the response network differentiating the macrophage responses to virulent and attenuated *M. bovis* strains [30].

### Role of cytokine-mediated signalling

GO enrichment analysis of the core response genes highlighted the importance of cytokine signaling pathways. Cytokine and chemokine-encoding genes were upregulated in cells infected with both morphotypes, and generally with larger fold changes for MAB-S-infected cells (Additional file 5: Table S2). A central role for cytokine-mediated signalling was also highlighted in a recent study comparing the THP-1 transcriptional responses to different MTB strains [16]. Given that differing cytokine responses have been reported between MAB-S and MAB-R morphotypes [30–32], we also measured their levels in culture supernatants.

**Fig. 4:**
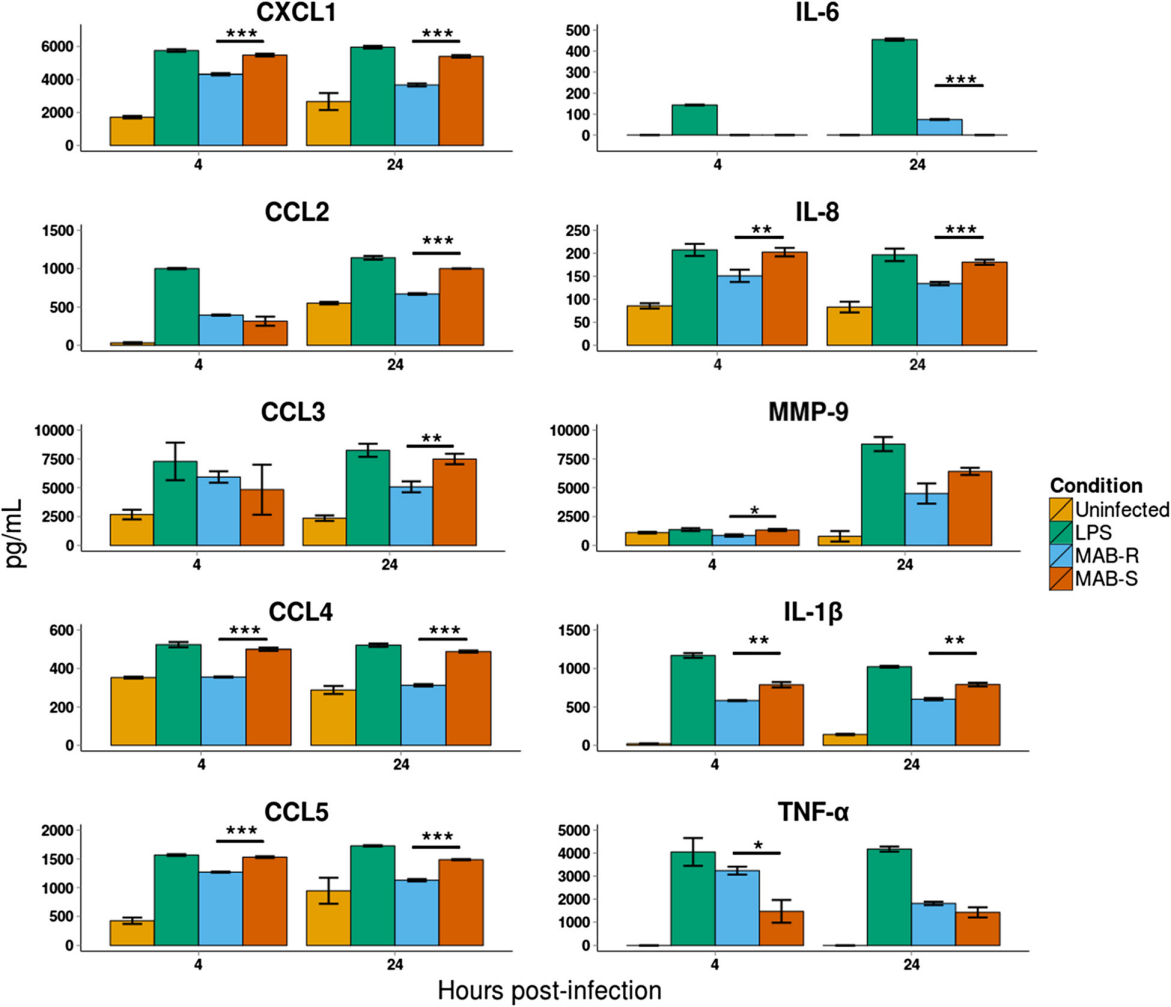
Measurements of cytokines and related proteins in cell supernatants. Increased production of pro-inflammatory cytokines and chemokines were observed in infected relative to uninfected cells. Additionally, morphotype-specific variations in the levels of several pro-inflammatory cytokines and chemokines were observed, with higher levels generally detected in MAB-S-infected cells. *Asterisks* indicate the level of statistical significance for morphotype-specific responses: * *P* ≤ 0.05, ** *P* ≤ 0.01, *** *P* ≤ 0.001

Clear morphotype-specific differences in cytokine levels were observed, with MAB-S eliciting a broadly stronger response (Fig. 4). We detected higher levels of TNF-α in culture supernatants of MAB-R-infected cells, consistent with previous reports that MAB-S inhibits its induction [8, 9, 33].

Both morphotypes elicited poor *IL-6* responses in the early stages of infection, with slightly increased induction at 24 hpi in MAB-R-infected cells (Fig. 4). The *IL-1β* and *IL-8* responses were found to be more robust, with higher levels detected in response to MAB-S infection (Fig. 4). The latter result is in contrast to a previous study in epithelial cells, in which MAB-R variants elicited increased expression of *IL-8* and *HβD2* [32].

Chemokines are a family of small molecular mass (8–14 kDa) chemotactic cytokines that shape the host immune responses to both rapidly and slowly growing mycobacteria [34–36]. Both MAB-R and MAB-S induced the transcription of a number of CC-chemokine genes, including *CCL3*, *CCL4*, and *CCL19*, which are known to be potent leukocyte activators and chemo-attractants with important roles in granuloma formation [36, 37]. Measurement of the protein levels of *CCL3* and *CCL4* in the culture supernatants of macrophages infected with MAB-R or MAB-S revealed them to be significantly higher in the latter (Fig. 4).

The convergence of these induced pro-inflammatory signalling pathways on the activation of the transcription factor NF-kB [37] is consistent with the upregulation of genes encoding the NF-kB complex by both morphotypes, including *NFKB1*, *NFKB2*, *REL*, *RELA* and *RELB*.

We also found that *MMP-9*, a metalloprotease involved in the remodeling of the extracellular matrix during the early events of granuloma formation [38], was detected in the cell supernatants in response to infection by both morphotypes (Fig. 4). *MMP-9* functions in conjunction with numerous cell-surface adhesion molecules that were found to be upregulated at the transcriptional level, including *ITGA1*, *ITGA6*, *TNFAIP6*, *CD44* and *ICAM1*, which plays a role in mycobacterial cell invasion [39].

### Morphotype-specific transcriptional responses

**Fig. 5:**
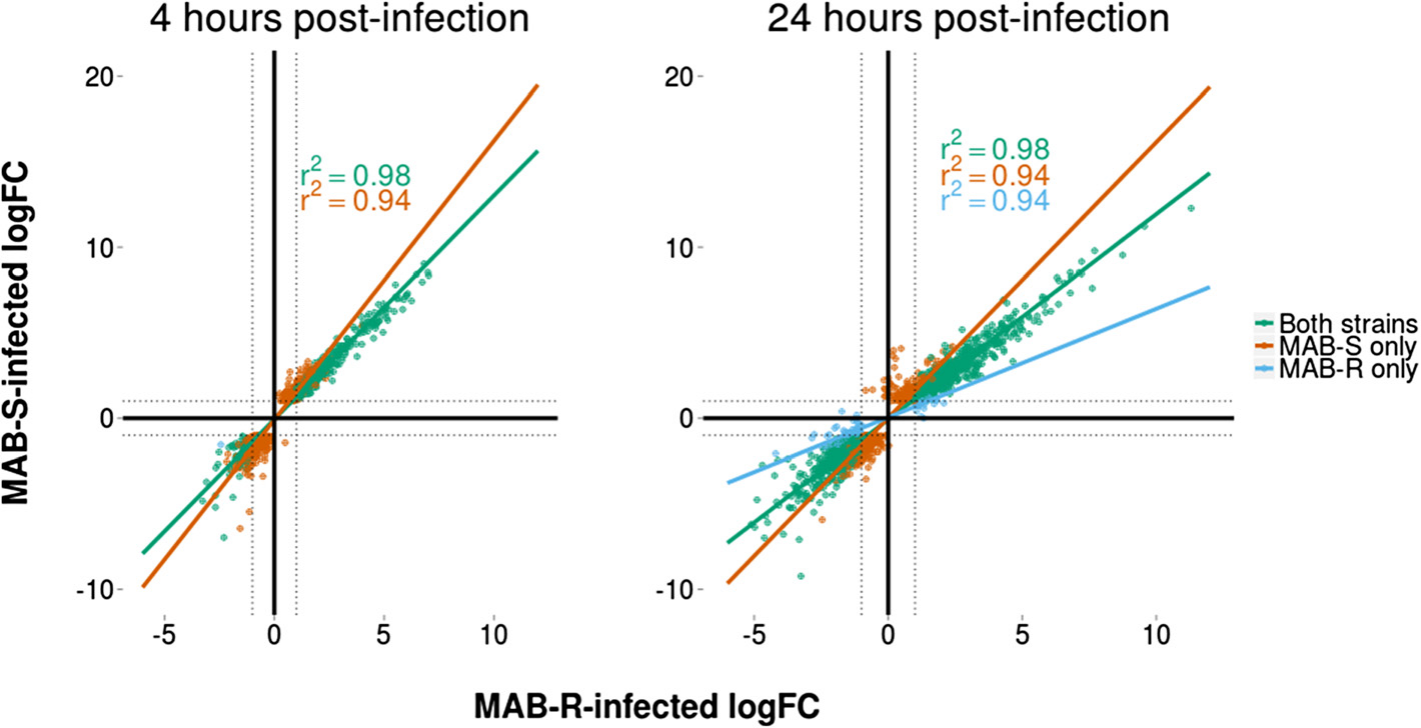
Correlation of gene expression between MAB-R- and MAB-S-infected cells. The log fold change (logFC) values of all DE genes are shown for cells infected by both strains at 4 hpi (**a**) and 24 hpi (**b**). All groups of genes (DE in response to both strains, DE in response to MAB-R only, DE in response to MAB-S only) show high levels of correlation (*r* ^2^ ≥ 0.94, *P* values all <2.2e-16), indicating the strong similarity of the transcriptional responses of macrophages to both strains

Although MAB-S elicited a more robust transcriptional response, a direct comparison between MAB-R- and MAB-S-infected cells showed the expression levels (log_2_ fold changes) of DE genes to be highly correlated (*r*^2^ ≥ 0.94, *P* values <2.2e-16) across all comparable groups (DE in both cases, DE in response to MAB-S only and DE in response to MAB-R only) (Fig. 5). The high level of correlation holds at both 4 and 24 hpi, and suggests that MAB-R and MAB-S-infected macrophages respond in a broadly similar manner. Correspondingly, just 6 and 34 DE genes were identified in a direct comparison of MAB-S and MAB-R-infected cells at 4, and 24 hpi, respectively. However, an analysis of this subset of genes suggests the potential for intriguing differences at the host-pathogen interface.

For example, the genes encoding Lysosomal-Associated Membrane Protein 3 (*LAMP-3* or *CD63*), Solute Carrier Family 7A member 8 (*SLC7A8*) and metallothionein 2A (*MT2A*) all show significantly increased expression in response to MAB-S relative to MAB-R. LAMPs are recruited to the mycobacterial phagosome and LAMP-3 is often used as a marker of phagosomal fusion. Studies in *M. avium* have shown that GPLs elicit a delayed phagosomal maturation in a mannose receptor (MR) dependent manner [6]. Blocking or limiting of MR expression was found to increase phagosome-lysosome fusion, as indicated by a significant increase in the number of lysosomal marker CD63-positive *M. avium* phagosomes [6]. The GPLs produced by MAB are thought to be relatively inert, however GPLs mask underlying cell wall mannosides and mannose-binding lectins bind more strongly to MAB-R than to MAB-S variants [7]. MAB MR-mediated phagosome delay could therefore explain the lower levels of *LAMP-3* expression detected in MAB-R-infected relative to MAB-S-infected cells.

*SLC7A8*, a member of the SLC7A family of cationic amino acid (y+) transporters, is also upregulated in response to MAB-S relative to MAB-R. The SLC7A family transporters are responsible for selective arginine uptake and the provision of arginine to various intracellular pools [40]. Arginine is the sole substrate for the inducible nitric oxide synthase (iNOS)-catalysed formation of nitric oxide (NO), which is utilized by activated macrophages to eliminate mycobacteria [41]. Altered *SLC7A8* suggest the potential for differing localized iNOS activity, with consequences for the production of NO and the extent of phagosome maturation between MAB-R- and MAB-S-infected cells.

Finally, our observation of increased expression of the *MT2A* gene in response to MAB-S relative to MAB-R is noteworthy. Metallothioneins are a family of membrane-bound proteins with important roles in the storage of heavy metals, including zinc [42], and it has been shown that zinc accumulates in the mycobacterial phagosome [43]. During the course of MTB infection, however, MTB pumps zinc out of the phagosome and the resultant increase in cytosolic free zinc induces increased the expression of zinc storage metallothionein (MT) genes, including *MT2A* [44]. Our finding of increased expression of *MT2A* in MAB-S-infected cells may reflect a more advanced level of phagosome maturation compared with MAB-R-infected cells, resulting in differing levels of free zinc metal in the corresponding cells.

### Integration of miRNA expression reveals networks of predicted targets among core response genes

**Fig. 6:**
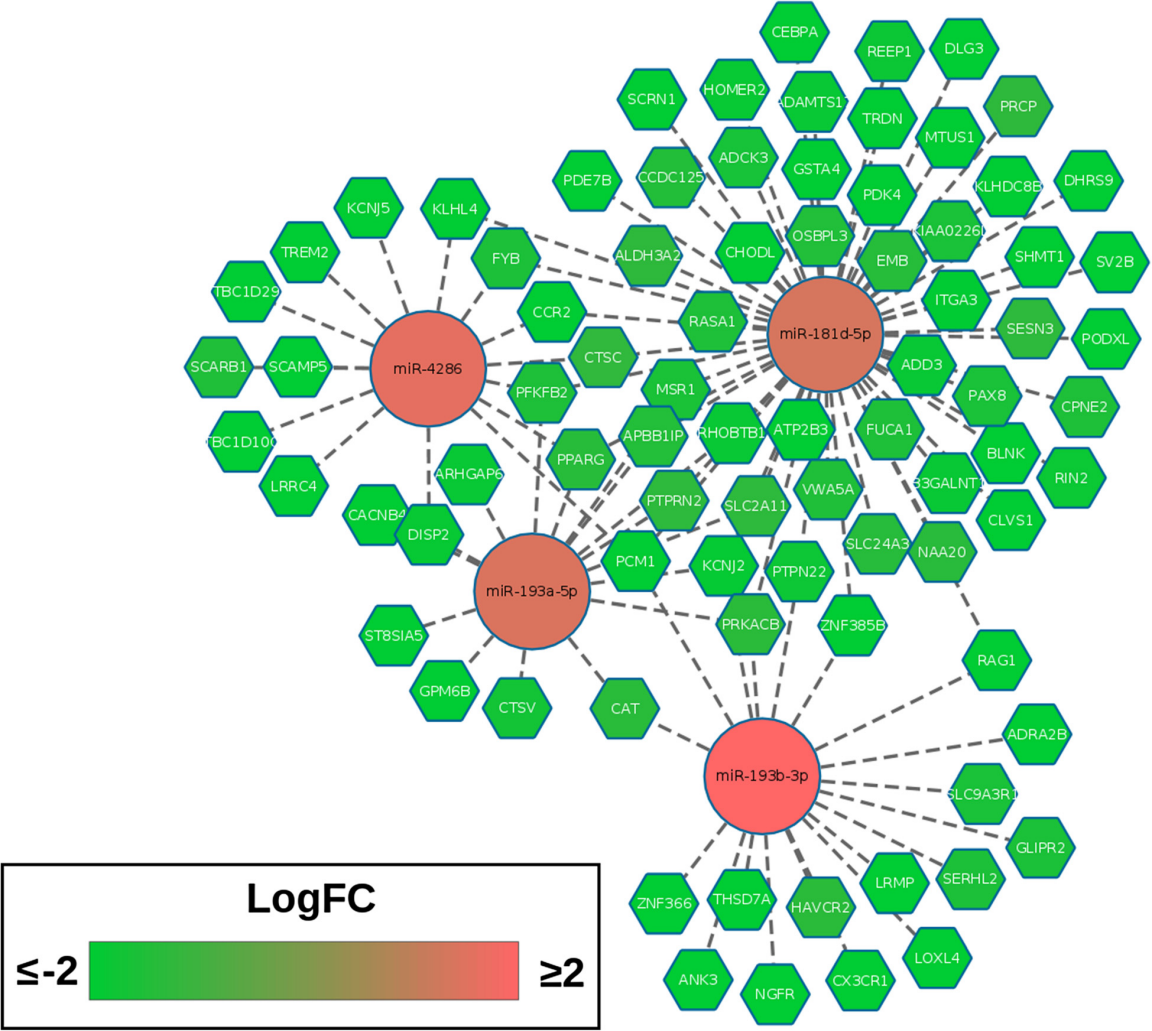
Integration of miRNA expression patterns with core response genes. Four of the top five ranked DE miRNAs from ToppMiR form an interconnected network of predicted interactions with target mRNAs of the core response network. Central to this network is miR-181d, which is regulated at 24 hpi in response to both MAB-R and MAB-S, and is predicted to interact with 57 core mRNAs showing inverse expression patterns. LogFC indicates the log fold change relative to uninfected cells

We found a total of 45 miRNAs to be DE at 24 hpi, 11 of which were common to both MAB-S- and MABS-R-infected cells (Additional file 9: Table S4). To study the functional significance of these shared DE miRNAs, we integrated their expression profiles with those of the core response genes and with miRNA target predictions using ToppMiR [45]. ToppMiR assigns a score to each miRNA given the functional relevance of its predicted targets (see Methods). Given that miRNAs predominantly decrease the expression of their targets [46], we looked for predicted interactions associated with inverse correlations in miRNA and mRNA expression levels. Four of the top five ranked miRNAs were found to comprise a cluster of predicted interactions with shared target mRNAs (Fig. 6). Central within the cluster, and up-regulated in both MAB-S and MAB-R-infected cells at 24 hpi, is miR-181d, with 57 predicted interactions (Fig. 6). miR-181d belongs to the miR-181 family, which mediates inflammatory responses through the regulation of PI3K and NF-κB signaling [47], and it has been shown to be upregulated in dendritic cells upon infection with MTB [48].

miR-193a-5p and miR-193b-3p are also both up-regulated at 24 hpi, and form part of the core network (Fig. 6). Increased expression of miR-193a-5p has been reported in patients with pulmonary TB as compared with healthy controls [49]. miR-193b-3p expression during mycobacterial infection has not been reported, however a previous study identified miR-193b-3p as one of the top circulating miRNA in mice exposed to gram-positive bacterial infection, with the potential to differentiate gram-positive from gram-negative infections [50]. miR-4286 has been shown to be downregulated upon the expression of the MTB latency-associated antigen Hsp16.3 [51], suggesting that its presence may be conducive towards productive infection.

miR-146, which is induced by variety of bacterial pathogens including *M. avium* [50, 52–55], as part of the antimicrobial response in macrophages, was induced exclusively in MAB-S-infected cells at 24 hpi. Other miRNAs specific to MAB-S-infected cells have also been found to differ in their expression levels between different strains of MTB (miR-99b and miR-140) or between latent and active tuberculosis (miR-877) [54]. The role of a subset of DE miRNAs in the disease progression of cystic fibrosis has also been described. Both the 5p and 3p arms of miR-126 were significantly down-regulated in MAB-S-infected cells compared to uninfected controls by 24 hpi. This miRNA has been shown to be differentially regulated in CF versus non-CF airway epithelial cells, and is thought to play an important role in regulating innate immune responses in the CF lung [55]. It has been hypothesized that miR-126 dysregulation in the CF lung may engender a TLR hyporesponsive state, which renders it less able to provide a rapid and robust immune response to aggravating infections [56].

## Conclusions

A robust core pattern of gene expression was observed upon infection with both MAB-S and MAB-R variants compatible with a TLR2 initiated signaling cascade, leading to the activation of nuclear factor kappa B (NF-kB) and resulting in a pro-inflammatory cytokine response. A network analysis highlighted the importance of type-I-Interferon and cytokine-mediated signaling to the core response, and identified three key hub transcription factors (*STAT1*, *SRC* and *EGR1*). IFN-α and IFN-β were not reported among the DE genes in our dataset, suggesting that, despite the strong activation of the Type-I- Interferon pathway, other mechanisms may interfere downstream in the cascade of the events that lead to the IFN production. In particular, we noticed that *TMEM173*, coding for the STimulator of INterferon Genes (STING), a downstream signaling adaptor required by RLRs [57, 58–60], was significantly down regulated in macrophages infected by both MAB variants. Indeed, it has been reported that MTB induces IFN production during macrophage infection via the activation of a *STING*/*TBK1*/*IRF3* signaling axis [61, 58].

The cytokine response was found to be pronounced at both the RNA and protein levels, with the latter also revealing morphotype-specific abundances and higher levels of key signaling molecules in response to the MAB-S morphotype. Given the potential of MAB to form granulomatous lesions [63, 64], it is likely that the secretion of these chemokines could help to create an early pro-inflammatory cell-rich niche in vivo, which facilitates the clearance of the bacilli. Upregulation of *DDX58* and *IFIH1* genes, coding for the RIG-I and MDA5 proteins respectively, suggest a host response to the leakage of bacterial nucleic acids into the cytosol. RIG-I signalling was seen to be over-represented in a recent transcriptomics study of *M. bovis*-infected bovine MDM [62]. The host miRNA response to MAB shows clear parallels with the responses observed to other mycobacterial pathogens, and appears to become more robust upon the adaptation of MAB to its host during the later stages of infection.

A number of previous studies have looked at the transcriptional differences between related strains of MTB with differing virulence and cell wall properties [29, 64, 66] and the results paint a complex picture. A study comparing MTB and cell wall deficient (CWD) MTB found the CWD forms stimulated lower levels of macrophage activation relative to the wild-type [63]. Another study involving MTB clinical strains CDC1551 and the hyper-virulent HN878 strain identified HN878 as eliciting a weaker early response, which then strengthened over time [64]. In both studies, host gene expression differences could be identified, including those encoding products involved in lipid metabolism and autophagy [64]. These studies are compatible with our finding of a poor early response to the more virulent MAB-R, relative to MAB-S, and the limited differences that separate them are enriched in genes associated with phagosome maturation. The results may point to differences in the manner that MAB-R is processed in the host macrophage relative to MAB-S, as previous work has shown that these morphotypes can reside in phagosomes of differing morphologies [9]. Differences in the induction of phagosomal maturation by rough and smooth variants, with the latter displaying a lesser capacity to inhibit phagosome maturation, have also been reported for MTB [65]. The potential for MAB-R to produce cords during infection could offer an explanation as to the potential for differing levels of phagosome maturation [66].

It is prudent, however, to interpret the limited number of transcriptional differences between morphotypes in the context of the high levels of correlation which were observed overall in expression patterns of DE genes. In the absence of functional data, it is not possible to determine the extent to which the subset of morphotype-specific DE genes contribute towards differing immune outcomes. Recent RNA-seq studies have questioned the role of such strain-specific transcriptional differences in eliciting differing immune responses. A large study comparing core response of THP-1 cells to infection with a wide variety of MTB strains [67], for example, found such differences difficult to link confidently to differing immune responses.

In summary, we find that the overall early responses of THP1-derived macrophages to MAB-S and MAB-R to be highly similar at the level of transcription, with the small numbers of differences having the potential to presage differing fates for the morphotypes later in the infection cycle. Our data should provide a platform for future studies in the functional genomics of host response to the R and S forms of MAB.

## Methods

### Bacterial strains and growth conditions

Isogenic Smooth and Rough variants of *M. abscessus* ATCC 19977-IP [11, 12] were used throughout this study. Mycobacterial strains were grown in Middlebrook 7H9 broth (Difco, BD Bioscience) supplemented with 0.2 % glycerol and albumin-dextrose complex (ADC, consisting of 0.5 % bovine serum albumin (BSA), fraction V, 0.085 % NaCl, and 0.2 % glucose) to a final concentration of 10 %, at 37 °C with shaking at 200 rpm.

Bacteria were harvested during the logarithmic growth phase, centrifuged at 4230 × *g* for 15 mins and subsequently washed twice by centrifugation at 4230 × *g* for 15 mins in phosphate buffered saline (PBS). To eliminate clumps of bacteria, after washing, cells were shaken with glass beads for 15 mins and then resuspended in PBS. The number of microorganisms was assessed by plating 10-fold dilutions of the bacterial suspension, in duplicate, on Middlebrook 7H11 agar (BDSciences), supplemented with 0.5 % glycerol and 10 % ADC, and CFU were counted after 1 week of incubation at 37 °C. At this stage, the number of bacteria at an OD_600_ of 3 was 2.6 × 10^9^ colony-forming units [CFU]/mL for MAB-R and 1.5 × 10^9^ CFU/mL for MAB-S.

Aliquots were kept frozen at −80 °C. To determine the survival rate of microorganisms after freezing, one week after stocks were prepared, 3 aliquots for each strain were defrosted and the number of microorganisms was assessed by plating 10-fold dilutions of the bacterial suspension, in duplicate, on Middlebrook 7H11 agar (BD Science). CFUs were counted after 1 week of incubation at 37 °C. At this stage, the number of bacteria at an OD_600_ of 3 was 2.4 × 10^9^ CFU/mL for MAB-R and 1.5 × 10^9^ CFU/mL for MAB-S.

The remaining aliquots were kept frozen at −80 °C until use. For each experiment, an aliquot of the bacteria was thawed, diluted in RPMI 1640 to a final OD_600_ of 0.125 for MAB-R and OD_600_ of 0.25 for MAB-S (1 × 10^8^ CFU/mL) and, immediately before use, was passed through a 29 gauge syringe to obtain a predominantly single-bacterial-cell suspension.

### Infection of THP-1 derived macrophages

THP-1 cells were obtained from the American Type Culture Collection (ATCC, TIB-202). The cells were grown in suspension in 75 cm^2^ flasks in RPMI medium supplemented with 10 % heat-inactivated fetal bovine serum (FBS, Life technologies), 1 % penicillin/streptomycin (Life technologies) and 2 mM L-Glutamine (Sigma) at 37 °C in a humidified CO_2_ incubator. Antibiotics were used in the growth medium of THP-1 cells to avoid contamination of the propagating cells.

To differentiate THP-1 cells into macrophages, the cells were seeded in 21 cm^2^ tissue culture dishes (CellStar) at a density of 10^7^ cells/dish and incubated with 25 ng/mL of phorbol 12-myristate 13-acetate (PMA, Sigma) for 24 h. Before the infection, the medium was replaced with fresh RPMI/10 % FBS/2 mM L-Glutamine medium without antibiotics and cells were infected with mycobacteria to achieve a multiplicity of infection (MOI) of 10:1 (bacteria:macrophages). Non-infected control macrophages received culture media only. Both not-infected and infected macrophages were prepared in triplicate in 21 cm^2^ tissue culture dishes and incubated at 37 °C.

After 1 hpi, the infected macrophages were washed with sterile PBS to remove extracellular mycobacteria and re-incubated until 4 or 24 hpi in culture media supplemented with 60 μg/mL of amikacin (Sigma) at 37 °C 5 % CO_2_, until the macrophages were harvested. This was necessary to inhibit the growth of any remaining extracellular bacteria. In preliminary experiments, we confirmed that, at this concentration, amikacin did not influence intracellular viability of both strains of MAB (data not shown).

At 1, 4 and 24 hpi, cells were washed with sterile PBS and lysed with PBS/0,01 % Triton X-100 (Sigma), and the number of intracellular bacteria was determined by plating 10-fold serial dilutions of cell lysates on solid medium. Viability of MAB-infected macrophages was evaluated by Trypan blue (Sigma) exclusion.

### RNA extraction

At 1, 4 and 24 hpi, infected macrophages were washed twice with PBS and homogenized by adding Trizol reagent (Sigma). All the aliquots were stored at −80 °C until required for RNA extraction.

RNA extraction was performed by adding 200 μL of chloroform (Sigma) to each sample and mixing by vortexing for 60 s. The solution was then centrifuged for 15 mins at 12,000 × *g* at 4 °C. The aqueous phase was removed, transferred to a separate tube containing 500 μL of isopropanol (Sigma), gently mixed and centrifuged for 10 mins at 12,000 × *g* at 4 °C. The supernatant was discarded, the RNA pellet was resuspended in 1 mL of 75 % Ethanol, mixed by vortexing and centrifuged 5 mins at 7500 × *g* at 4 °C. After discarding all the supernatants, the RNA was transferred to a miRNeasy mini kit column (Qiagen, Ltd.) and further purification steps were conducted according to the manufacturer’s instructions. In addition, the RNA was subjected to an on-column DNase treatment step (Qiagen, Ltd.) to remove any DNA residues that could affect the downstream reaction.

RNA quantity and quality was assessed using a NanoDrop^TM^ 1000 spectrophotometer (Thermo Fisher Scientific, Inc.) and on an Agilent 2100 Bioanalyzer using an RNA 6000 Nano LabChip kit (Agilent Technologies, Ltd.). All samples displayed a 260/280 ratio greater than 2.0 and RNA integrity numbers (RIN) greater than 7.5.

### Messenger RNA-seq libraries preparation and sequencing

In total, 27 strand-specific RNA libraries for high-throughput sequencing were prepared (three biological replicates for each treatment) using the TruSeq Stranded mRNA Sample Preparation Kit according to the manufacturer’s protocol. Briefly, the poly-A containing mRNA molecules were purified from 4 ug of total RNA (extracted from non-infected and infected samples) using poly-T oligo-attached magnetic beads. The mRNA was then fragmented and the first-strand cDNA was synthesized using reverse transcriptase (SuperScript II, Invitrogen) and random hexamers. Second cDNA synthesis was performed by removing the RNA template and synthesizing a replacement strand, incorporating dUTP in place of dTTP, to generate double-stranded (ds) cDNA. dsDNA was then subjected to the addition of “A” bases to the 3′ ends and ligation of the barcoded TruSeq adapters. Amplification of fragments with adapters ligated on both ends was performed by PCR using the primer cocktail supplied in the kit. Final libraries were analysed on an Agilent 2100 Bioanalyzer using the Agilent DNA 1000 chip (Agilent Technology) to confirm that the fragment size range was ~200–260 bp. All libraries were then quantified using a Qubit fluorometer and Qubit double stranded DNA High Sensitivity kit (Invitrogen). Libraries were loaded at a concentration of 10 pM onto the flow cell and were single-end sequenced on an Illumina HiSeq 2000.

### Small RNA-seq libraries preparation and sequencing

Twenty−Five small RNA-seq (sRNA-seq) libraries were prepared using the Illumina TruSeq Small RNA Sample Preparation Kit, according to the manufacturer’s protocol, using 1 μg of total RNA (extracted from non-infected and infected samples). Briefly, RNA adapters were ligated to the 5′ and 3′ ends of the small RNA molecules, followed by reverse transcription and 11 cycles of PCR amplification by using indexed primers.

The libraries were size-selected on a 6 % polyacrylamide gel for the desired size range of 147–157 nt, and then purified from the gel. The molarity and size of finished miRNA-seq libraries were quantified on an Agilent 2100 Bioanalyzer using an Agilent High Sensitivity DNA chip (Agilent technologies). Libraries were loaded at a concentration of 10 pM onto the flow cell and were single-end sequenced on Illumina HiSeq 2000.

### Cytokine measurements

Supernatants from THP-1-derived macrophages stimulated with MAB-R, MAB-S, or LPS, and from uninfected macrophages, were collected at 1, 4 and 24 hpi and stored in aliquots at −80 °C until use.

Cytokine measurements were performed using the RayBio® Quantibody Human Cytokine array, according to the manufacturer’s instructions (RayBiotech, Inc.). A GenePix 4000B scanner (Axon) with GenePix software was used to collect fluorescence intensities. Cytokine concentrations were determined by RayBiotech Inc. using their Quantibody service.

### Read processing and alignment

Quality assessment of reads was carried out using FastQC [68] to assess the distribution of phred quality scores and mean percentage GC content across each read. Adapters were trimmed from the 3′ ends of reads using Fastx-toolkit [69], and the reverse complement of each trimmed read was obtained to account for the TruSeq Stranded library generation process. Trimmed and reverse-complemented reads were aligned to the the latest human genome assembly from the Genome Reference Consortium (version GRCh38) using the Star universal RNA-seq aligner [70] (mRNA libraries) or bowtie [71] (miRNA libraries). Aligned reads were stored in the SAM file format. sRNA-seq libraries with low read coverage (<200,000 reads aligned) were discarded from further analysis.

### Statistical analyses

HTSeq [72] was used to count the number of reads mapping unambiguously to the sense strand of each annotated gene. In the case of poly-A selected libraries, only reads mapping to protein-coding genes were counted. Read counts were passed to the Bioconductor package edgeR [13], and genes found to be expressed in at least 3 libraries (counts per million [CPM] ≥ 1 for mRNA libraries and CPM ≥10 for miRNA libraries) were retained for downstream analyses. A negative binomial generalized linear model (GLM) was fitted to each gene using edgeR [13]. Multiple testing correction was applied using the approach of Benjamini and Hochberg [73], and differentially expressed (DE) genes were identified as those with FDR <0.05 and an absolute log_2_ fold change in abundance of at least 1. For hierarchical clustering of libraries, variance stabilisation of read counts was first performed using DEseq [74]. The 50 genes with highest variance were identified using the genefilter package in R, and the mean variance-stabilised expression values of biological replicates were used to perform hierarchical clustering.

### Core response genes

The log_2_ fold change values at 1, 4, and 24 hpi were used to match DE genes to temporal response profiles using STEM. Profiles with a high correlation (*r* > 0.8) were merged, and profiles from MAB-R- and MAB-S-infected cells were merged if they had a significantly higher overlap of genes than expected due to chance (*P* < 0.05). Merged clusters of genes comprised the core response. GO enrichment analysis and TFBS enrichment analysis of core response genes were carried out with InnateDB [22], using data from the cisRED database [17]. Analysis of inter-connectivity between enriched GO terms was performed using the ClueGO app for Cytoscape [15].

### Network construction and analysis

Validated interactions between core response genes were retrieved from the InnateDB database [22], and visualised as a network using Cytoscape [75]. Topological parameters, including degrees of connectivity (DOC) and betweenness centrality (BC), were calculated and a power law was fitted to the distribution of DOC values using the NetworkAnalyzer app for Cytoscape [75]. Random networks of an equivalent size were generated according to the Erdos-Renyi model using the iGraph package [76] for R. The clustering co-efficient (*C*) and average shortest path lengths (*L*) of random networks were compared with those observed for the actual core response network. The small-world coefficient (σ) was calculated as [(*C*÷*C*_*R*_) ÷ (*L*÷*L*_*R*_)], where *C*_*R*_ and *L*_*R*_ are the mean clustering co-efficient and average shortest path length, respectively, of equivalently-sized randomly-generated networks [26]. To compare the DOC and BC values of hubs and bottlenecks to those expected to occur by chance, the core network was randomly-rewired 1000 times, and the DOC and BC of the hub and bottleneck genes were calculated each time.

### miRNA network integration

Common DE miRNAs and core response mRNAs were uploaded to ToppMiR [45] to identify potential interactions. Default settings were used, and miRNAs were ranked according to the functional relevance of their predicted target mRNAs. The expression profiles (mean log_2_ fold changes at 24 hpi) of both mRNAs and miRNAs were further integrated to refine interaction predictions, and those with inverse correlations were identified.

## Availability of supporting data

RNA-seq and sRNA-seq data have been deposited at GenBank under the accession number GSE72822.

## Additional files

Additional file 1: Figure S1. Viability of intracellular MAB. Line plots show the numbers of viable bacteria derived from infected cells, given as log10 colony forming units (CFU). The number of intracellular bacteria was determined by plating 10-fold serial dilutions of cell lysates on solid medium. No significant differences were observed between MAB-S and MAB-R intracellular growth from 1 – 72 hours post infection. (PNG 32 kb) Additional file 2: Figure S2. Macrophage death assay. Viability of MAB-infected macrophages was evaluated by Trypan blue exclusion. Bar plots show the percentage of dead macrophages in response to MAB-R and MAB-S infection, as well as among uninfected controls. No significant differences were observed in cell death between MAB-S and MAB-R infected cells from 1 – 24 hours post infection (hpi). At 48 and 72 hpi, a significantly higher proportion of macrophages were found to have died in response to MAB-R infection. (PNG 31 kb) Additional file 3: Table S1. Read mapping statistics for RNA-seq and small RNA-seq (sRNA-seq) libraries. For RNA-seq libraries, the numbers of reads mapping uniquely to the human reference genome are indicated. For sRNA-seq libraries, the numbers of reads mapping to mature miRNAs from miRBase are given. (XLSX 7 kb) Additional file 4: Figure S3. Multidimensional scaling (MDS) plot of libraries at the indicated time point post infection, based upon log Fold Change (logFC) values of expressed genes. Infected and uninfected libraries are observed to cluster discretely at each time point. (PNG 37 kb) Additional file 5: Table S2. Differentially expressed (DE) mRNAs identified in response to MAB-R and MAB-S infection. Genes were regarded as DE if they had an absolute Log2 Fold Change of at least 1, and an adjusted P-value (FDR) of less than 0.05. (XLSX 336 kb) Additional file 6: Table S3. Overlap of the core responses to MAB and MTB. 73 genes were identified in both datasets. “Induced” indicates that the gene shows an increase in abundance upon infection; “Repressed” indicates that the gene shows a decrease in abundance upon infection. (XLSX 8 kb) Additional file 7: Figure S4. Comparison of the core response network to 1,000 randomly rewired networks of an equivalent size. The percentage of genes at each indicated distance from the nearest hub gene is shown. (PNG 42 kb) Additional file 8: Figure S5. Betweenness centralities and degrees of connectivity of genes comprising the core response network. Hub and bottleneck genes are shown in red; other genes are shown in gray. Dashed lines indicate the mean values for the entire network. (PNG 69 kb) Additional file 9: Table S4. Differentially expressed (DE) miRNAs identified in response to MAB-R and MAB-S infection. miRNAs were regarded as DE if they had an absolute Log2 Fold Change of at least 1, and an adjusted P-value (FDR) of less than 0.05. (XLSX 7 kb)

## Abbreviations

DE: Differentially expressed
MAB-S: *Mycobacterium abscessus* Smooth morphotype
MAB-R: *Mycobacterium abscessus* Rough morphotype
hpi: Hours post-infection
STEM: Short Time-series Expression Miner
