# Supplementary figures and images for: High-throughput transcriptomics reveals common and strain-specific responses of human macrophages to infection with Mycobacterium abscessus Smooth and Rough variants
Source: BMC Genomics. 2015 Dec 9;16:1046. doi: 10.1186/s12864-015-2246-1 (PMC4674915; doi:10.1186/s12864-015-2246-1)

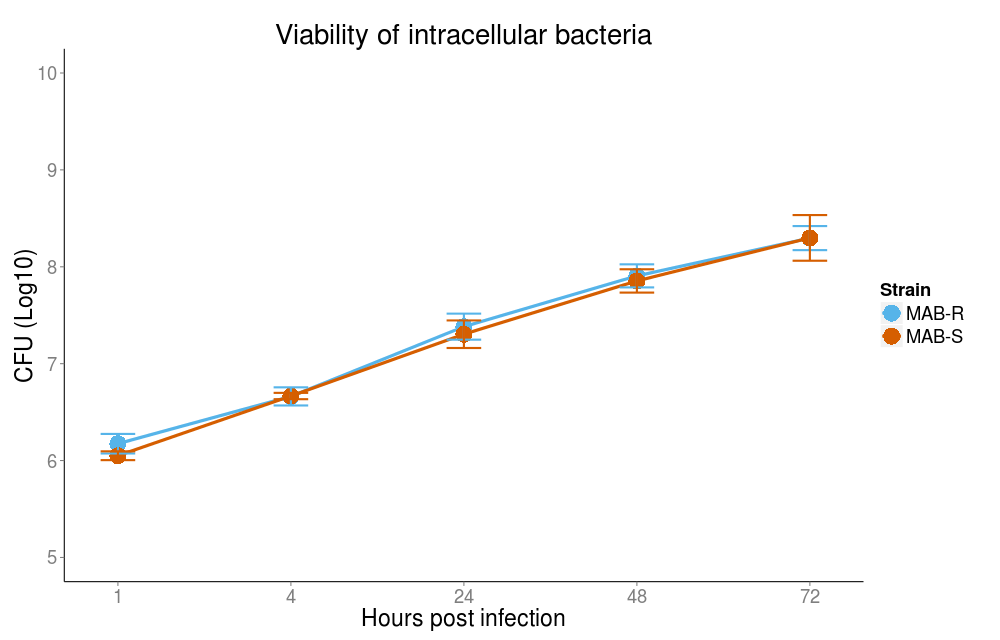

Supplement: Additional file 1: Figure S1. — Viability of intracellular MAB. Line plots show the numbers of viable bacteria derived from infected cells, given as log10 colony forming units (CFU). The number of intracellular bacteria was determined by plating 10-fold serial dilutions of cell lysates on solid medium. No significant differences were observed between MAB-S and MAB-R intracellular growth from 1 – 72 hours post infection. (PNG 32 kb) [file 12864_2015_2246_MOESM1_ESM.png]

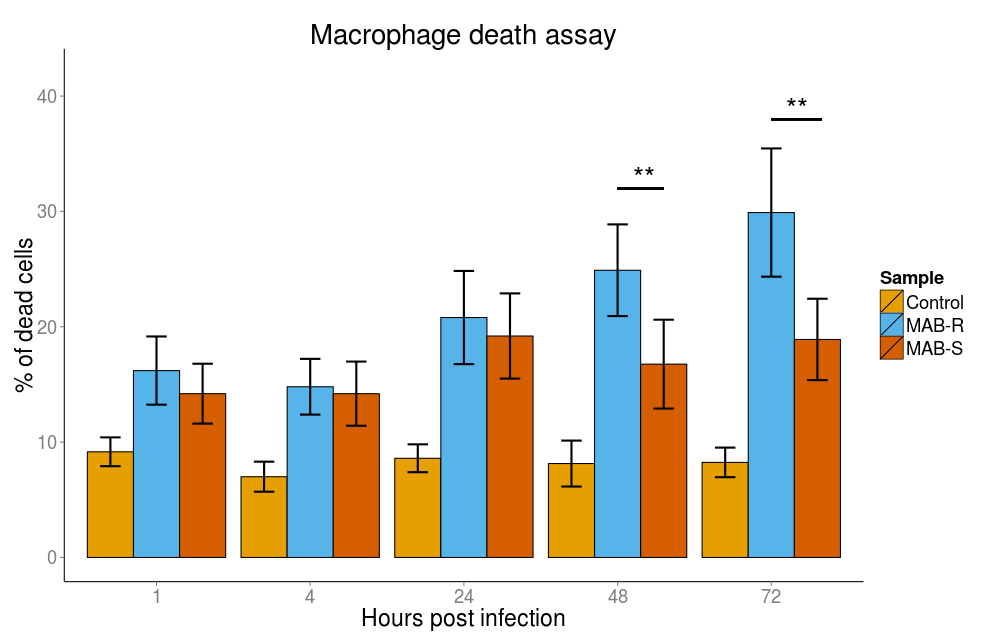

Supplement: Additional file 2: Figure S2. — Macrophage death assay. Viability of MAB-infected macrophages was evaluated by Trypan blue exclusion. Bar plots show the percentage of dead macrophages in response to MAB-R and MAB-S infection, as well as among uninfected controls. No significant differences were observed in cell death between MAB-S and MAB-R infected cells from 1 – 24 hours post infection (hpi). At 48 and 72 hpi, a significantly higher proportion of macrophages were found to have died in response to MAB-R infection. (PNG 31 kb) [file 12864_2015_2246_MOESM2_ESM.png]

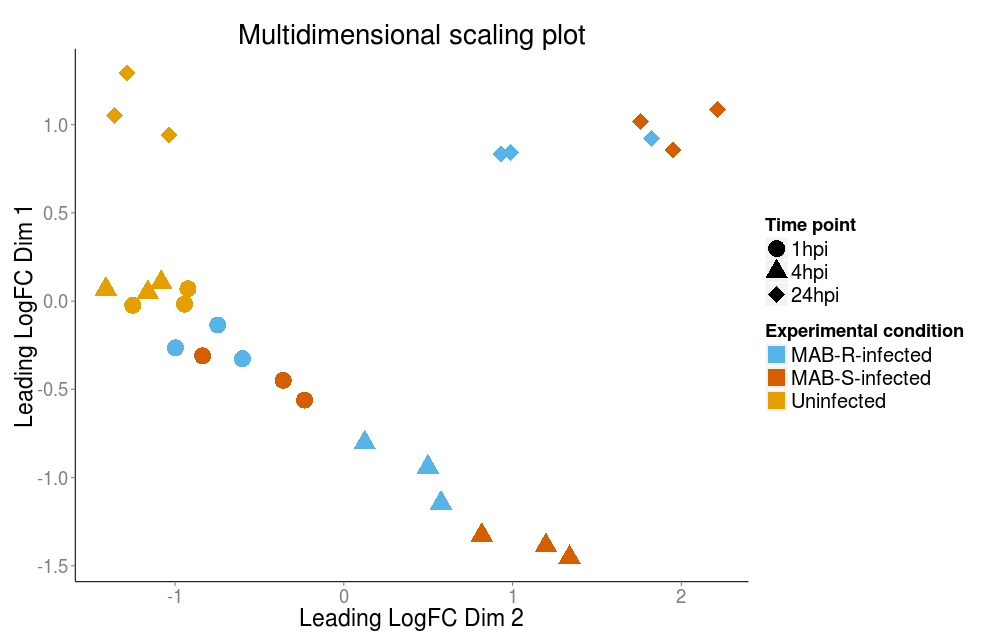

Supplement: Additional file 4: Figure S3. — Multidimensional scaling (MDS) plot of libraries at the indicated time point post infection, based upon log Fold Change (logFC) values of expressed genes. Infected and uninfected libraries are observed to cluster discretely at each time point. (PNG 37 kb) [file 12864_2015_2246_MOESM4_ESM.png]

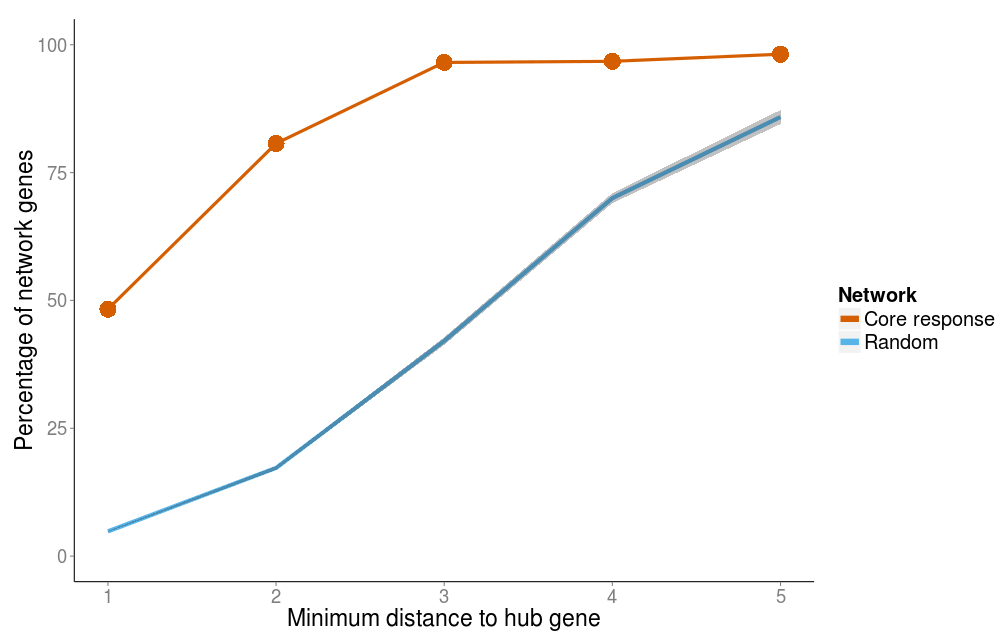

Supplement: Additional file 7: Figure S4. — Comparison of the core response network to 1,000 randomly rewired networks of an equivalent size. The percentage of genes at each indicated distance from the nearest hub gene is shown. (PNG 42 kb) [file 12864_2015_2246_MOESM7_ESM.png]

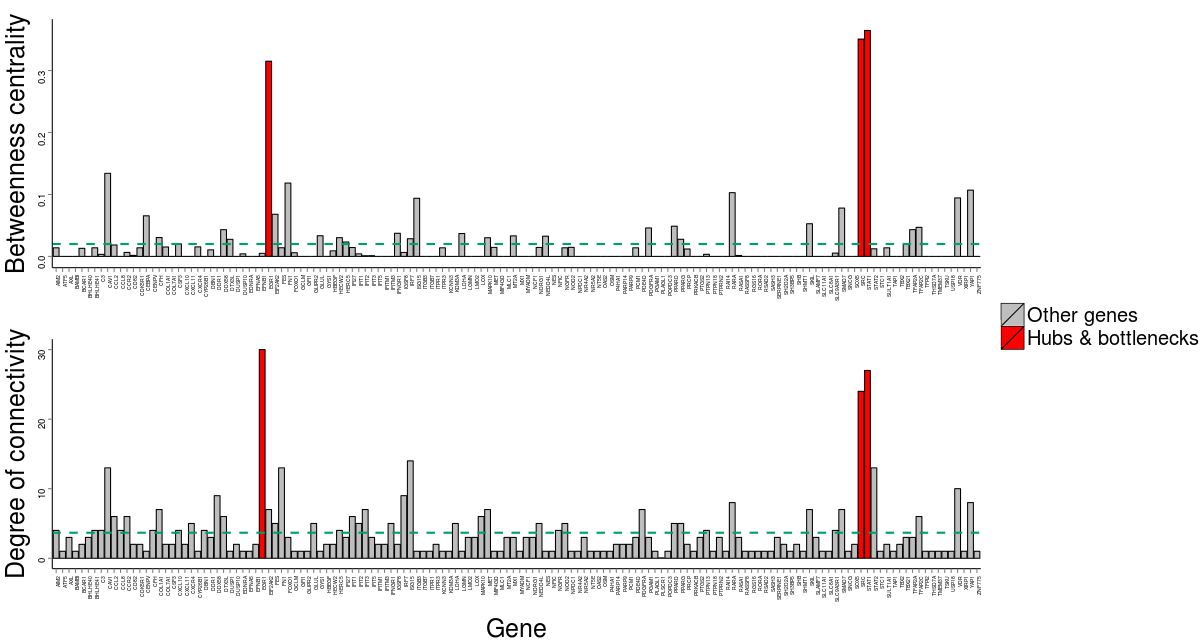

Supplement: Additional file 8: Figure S5. — Betweenness centralities and degrees of connectivity of genes comprising the core response network. Hub and bottleneck genes are shown in red; other genes are shown in gray. Dashed lines indicate the mean values for the entire network. (PNG 69 kb) [file 12864_2015_2246_MOESM8_ESM.png]
